# Population genomic monitoring provides insight into conservation status but no correlation with demographic estimates of extinction risk in a threatened trout

**DOI:** 10.1111/eva.13473

**Published:** 2022-09-04

**Authors:** William Hemstrom, Daniel Dauwalter, Mary M. Peacock, Douglas Leasure, Seth Wenger, Michael R. Miller, Helen Neville

**Affiliations:** ^1^ Department of Animal Science University of California Davis California USA; ^2^ Science Program Trout Unlimited Boise Idaho USA; ^3^ Department of Biology University of Nevada Reno Nevada USA; ^4^ WorldPop, Geography and Environmental Science University of Southampton Southampton UK; ^5^ Odum School of Ecology University of Georgia Athens Georgia USA

**Keywords:** evolutionary adaptation, genetic monitoring, hybridization, population viability analysis, RAD sequencing, salmonid

## Abstract

The current extinction crisis requires effective assessment and monitoring tools. Genetic approaches are appealing given the relative ease of field sampling required to estimate genetic diversity characteristics assumed related to population size, evolutionary potential, and extinction risk, and to evaluate hybridization with non‐native species simultaneously. However, linkages between population genetic metrics of diversity from survey‐style field collections and demographic estimates of population size and extinction risk are still in need of empirical examples, especially for remotely distributed species of conservation concern where the approach might be most beneficial. We capitalized on an exceptional opportunity to evaluate congruence between genetic diversity metrics and demographic‐based estimates of abundance and extinction risk from a comprehensive Multiple Population Viability Analysis (MPVA) in a threatened fish, the Lahontan cutthroat trout (LCT). We sequenced non‐native trout reference samples and recently collected and archived tissue samples of most remaining populations of LCT (*N* = 60) and estimated common genetic assessment metrics, predicting minimal hybridization with non‐native trout, low diversity, and declining diversity over time. We further hypothesized genetic metrics would correlate positively with MPVA‐estimated abundance and negatively with extinction probability. We uncovered several instances of hybridization that pointed to immediate management needs. After removing hybridized individuals, cautious interpretation of low effective population sizes (2–63) suggested reduced evolutionary potential for many LCT populations. Other genetic metrics did not decline over time nor correlate with MPVA‐based estimates of harmonic mean abundance or 30‐year extinction probability. Our results demonstrate benefits of genetic monitoring for efficiently detecting hybridization and, though genetic results were disconnected from demographic assessment of conservation status, they suggest reduced evolutionary potential and likely a higher conservation risk than currently recognized for this threatened fish. We emphasize that genetic information provides essential complementary insight, in addition to demographic information, for evaluating species status.

## 1 INTRODUCTION

The need for effective assessment and monitoring tools is ever more pressing given the current extinction crisis (Butchart et al., 2010; Ceballos et al., 2020; Hohenlohe et al., 2021). Genetic monitoring (a term we use generally to encompass both one‐time assessment and evaluation over time of various genetic metrics, but see Schwartz et al., 2006) may be particularly germane due to the relative ease of broad‐scale field sampling for evaluation of metrics assumed related to population size, such as allelic diversity or heterozygosity, when compared with more traditional methods of population monitoring (Frankham, 1995; Schwartz et al., 2006). Genetic information also uniquely provides the potential to shed light on other aspects of risk simultaneously (Carroll et al., 2018; Proença et al., 2017; Schwartz et al., 2006). For example, the same genetic samples could be used for assessing mechanisms of loss such as low effective population sizes (Frankham, 2015) and to gauge impending consequences of genetic decline over time such as reduced population fitness and evolutionary potential (Bijlsma & Loeschcke, 2011; Leroy et al., 2018; Reed & Frankham, 2003), and increased extinction risk (Saccheri et al., 1998). Furthermore, loss of genetic diversity over time (i.e., genetic erosion) can provide essential context for interpreting recent population dynamics (e.g., inferences of increased instability or isolation, Leroy et al., 2018) and management needs. Finally, for many species, genetic approaches enable simultaneous evaluation of an entirely separate but major conservation threat that may not be captured accurately by other means—that of hybridization with non‐native species (Allendorf et al., 2001; Dufresnes et al., 2019; Schwartz et al., 2006; Taylor et al., 2020).

Despite this promise, much of the rapid expansion of genetic monitoring has been in species for which noninvasive sampling (via genetic fingerprinting using hair, feathers or feces) enables direct estimates of abundance in targeted populations through individual identification or mark‐recapture (Carroll et al., 2018; Luikart et al., 2010; Schwartz et al., 2006), or for surveying genetic metrics to infer conservation risk (Schwartz et al., 2006; Shafer et al., 2015) often as proxies for but without accompanying demographic estimates (e.g., Osborne et al., 2012; Ottewell et al., 2016; Pavlova et al., 2017). For genetic metrics to be applied broadly and efficiently in lieu of traditional effort‐intensive demographic sampling requires further verification that commonly evaluated genetic proxies indeed provide meaningful characterization of information important for management and conservation, such as current population abundances and extinction probabilities. Efforts to link the two types of information, i.e., via spatially consistent and temporally stable ratios of genetic diversity to census size that could allow estimation of one to infer the other across populations, have long shown promise (e.g., estimating *N*_e_/*N*_c_, Bernos & Fraser, 2016; Ferchaud et al., 2016; Frankham, 1995), but strong observed temporal and among‐population variation (Ardren & Kapuscinski, 2003; Luikart et al., 2010; Palstra & Fraser, 2012; Ruzzante et al., 2016) suggests this approach still may be difficult to rely on in a general sense. Thus, linkages between sampled genetic indicators and demographic characteristics, adaptive capacity or evolutionary response and extinction risk are still elusive in many cases (Wang & Shaffer, 2017; Wood et al., 2016) and will benefit from large‐scale, survey‐style empirical examples (Ørsted et al., 2019). This is especially true for nonmodel organisms of conservation concern, where such real‐world examples may be particularly salient for evaluating when genetic (or genomic) assessment will be most applicable or where various complexities of analyses and nature might prevent clear interpretation (Shafer et al., 2015).

Here, we capitalized on an exceptional opportunity to evaluate congruence between genetic indicators of population size and evolutionary risk (genetic diversity and effective population size, respectively) attained from available survey‐style field collections, and demographic‐based estimates of abundance and extinction risk from a comprehensive population viability analysis applied across the historical range of a threatened fish in the inland United States. We first applied modern sequencing techniques to recently collected (~2015–2017) tissue samples to estimate common genetic monitoring metrics across most remaining conservation populations (*N* = 60) of the Lahontan cutthroat trout (LCT, *Oncorhynchus clarkii henshawi*). Importantly, we included reference samples (Ravagni et al., 2021) of both rainbow trout (*O. mykiss*) and Yellowstone cutthroat trout (YCT; *O. bouvieri*), trout not native to this region but which have been introduced to the LCT range and present a primary threat to the genetic integrity of LCT via introgression. Effective population sizes were interpreted cautiously due to reliance on small and multi‐cohort samples, as we discuss, but provide a standing gage of genetic health and evolutionary potential for LCT. We were also able to test for genetic erosion (Carroll et al., 2018; Hoban et al., 2014; Leroy et al., 2018) in numerous populations where archived samples enabled evaluation of change over time, in some cases over a period spanning several decades and characterized by intense drought. These analyses yielded the most comprehensive range‐wide and temporal assessment of population hybridization status, effective size, and genetic diversity for this threatened trout since its listing over five decades ago under the Endangered Species Act (see also Peacock & Kirchoff, 2007; USFWS, 1970).

We next looked for concordance between metrics of genetic diversity and demographically derived estimates of abundance and 30‐year extinction risk from a comprehensive, range‐wide Multiple Population Viability Analysis (MPVA) recently applied across LCT stream populations (Neville et al., 2019). Population Viability Analysis is a class of modeling approaches used to estimate population trajectories and extinction risks over given time frames (Beissinger & McCullough, 2002). MPVA is a novel approach to population viability analysis based on a Bayesian hierarchical modeling framework with linked observation, sampling, and population dynamics models (Leasure et al., 2019; Wenger et al., 2017). It has several desirable attributes uncommon in population viability analysis, such as using demographic field data directly and employing covariates reflecting environmental conditions to fit the observation and sampling submodels, that inform a population dynamic model enabling estimation of abundance and extinction risk across all populations simultaneously. The model applied here used over 30 years of field surveys representing all available demographic data for LCT (15,265 LCT captured during 3967 field surveys from 1980 to 2015) and annual estimates of non‐native trout abundance, stream temperature, riparian condition, and streamflow as field‐collected and remotely‐sensed covariates to estimate abundance and 30‐year extinction probabilities for each population (Leasure et al., 2019; Neville et al., 2019).

Based on formal management delineation of most of our sampled populations as “conservation populations” assumed to be uncompromised by introgression (USFWS, 2009) and earlier studies of selected populations (Amish et al., 2019; Neville et al., 2006; Peacock & Dochtermann, 2012), we expected populations of this threatened trout generally to show little evidence of hybridization with rainbow or Yellowstone trout but to be characterized by low genetic diversity and effective sizes. As well, given the isolated nature (Dunham et al., 1997) and volatile dynamics of LCT populations in particular (Platts & Nelson, 1988) and several extreme periods of drought within the time frame encompassing our temporal samples, we anticipated evidence of genetic erosion (Leroy et al., 2018), or loss of diversity over time. Finally, as observations from MPVA demonstrated a broad range of demographically derived estimates of abundance and extinction probability across LCT populations (Neville et al., 2019), we hypothesized populations with higher genetic diversity would have higher estimated abundance and lower 30‐year extinction probabilities. Our study provides an unusual ability to evaluate correlations between genetic and demographic metrics across the range of a threatened species and confirms both the promise and complexities of real‐world genomic assessment.

## 2 METHODS

### 2.1 Study system

**FIGURE 1:**
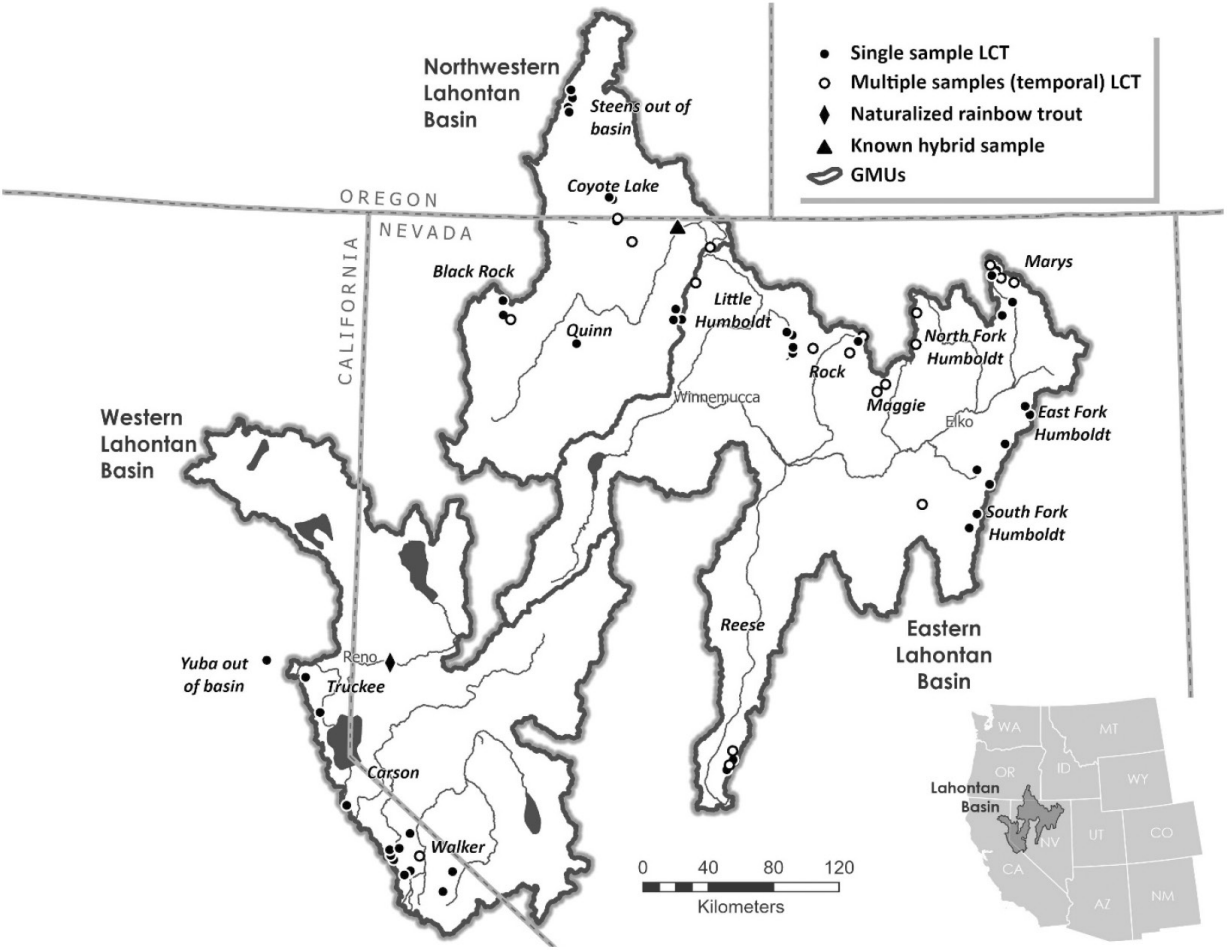
Map of sample sites from LCT populations in Nevada, California, and Oregon, United States, with single (filled circles) and temporal (open circles) samples denoted; samples from several out of basin transplanted populations are also indicated. Bold lines denote three Major Geographic Management Units for LCT (Western, Eastern, Northwestern), and major river basins are noted. Location of a reference sample of naturalized rainbow trout and a previously assessed hybrid sample are also shown.

The LCT persists in a highly dynamic and remote desert environment in the northern portion of the Great Basin Desert in the western United States (Figure 1). Remaining populations are found in mostly isolated habitats (Dunham et al., 1997) comprising less than 10% of the species' historical stream distribution (and <1% of its historical lake distribution, though we did not evaluate lake populations here, USFWS, 2009). Population isolation is partly a natural consequence of the long‐term drying of the Great Basin, as most streams originating in mountains now subside into desert playas (Grayson, 1993; Reheis et al., 2002), but it is greatly exacerbated by physical barriers such as dams, culverts and irrigation diversions as well as fragmentation due to flow modification and inhospitable temperatures in degraded habitats. Physical isolation of LCT populations has multiple impacts, including reducing abundances and restricting dispersal and gene flow as well as preventing maintenance of a migratory life history, wherein many individuals spawn in headwater streams but then move to productive seasonal habitats in larger mainstem rivers (Armstrong et al., 2021; Neville et al., 2006; Rieman & Dunham, 2000). Migratory behavior in salmonids provides increased reproductive capacity (from large, fecund migratory females, e.g., Ohlberger et al., 2020) and ecological connectivity to varied complementary and refuge habitats (Bisson et al., 2009; Dunham et al., 2002), both major components of resiliency now largely lost in LCT. Further, hybridization with non‐native trout across LCT's historical range is a primary threat to the genetic integrity of this and other cutthroat trout subspecies (Allendorf & Leary, 1988; Hitt et al., 2003; Muhlfeld et al., 2009). The multiple threats faced by LCT continue to impact this subspecies and are increasingly important to monitor in the future under climate change, which is already eroding and reducing aquatic habitat in this region (Schultz et al., 2017; Williams et al., 2020) and may increase the threat of hybridization (Muhlfeld et al., 2014, 2017).

### 2.2 Sampling

A total of 1336 samples of individual LCT were included in this study. Samples were compiled from available recent and archived LCT fin tissue samples from populations across the historical distribution of LCT including several out‐of‐basin samples of agency or conservation interest (Figure 1). Samples were collected by state agencies (Nevada Department of Wildlife, Oregon Department of Fish and Wildlife, and California Department of Fish and Wildlife) as part of on‐going assessment programs, or the authors (M. Peacock, H. Neville) for this or previous studies (Neville et al., 2006, 2016; Peacock et al., 2010; Peacock & Dochtermann, 2012; Peacock & Kirchoff, 2007). There is no unified sampling protocol for LCT, leading to somewhat sporadic temporal and spatial sampling across the range and differing methods of sampling within populations (e.g., number of sites per stream, number of electrofishing passes per site), but in all cases field sampling involved electrofishing age 1+ individuals across multiple (typically 3+) sections of each stream to ensure representation of genetic variability within each population and avoid sibling groups (Hansen et al., 1997). Where possible, field crews generally attempt to collect 20–50 individuals from each stream population although in many situations they do not encounter this minimum targeted number, leading to smaller sample sizes. Fin clips were dried and stored either in coin envelopes or on gridded chromatography paper (LaHood et al., 2008).

We included several reference samples to evaluate the threat of hybridization with both non‐native rainbow and YCT across all populations. For rainbow trout, we included a sample collected by the US Fish and Wildlife Service from a naturalized population in the Truckee River, a historical LCT spawning tributary to Pyramid Lake; a field population previously verified as introgressed with LCT using a diagnostic set of microsatellites (see Figure 1); and previously published whole‐genome sequencing data from a set of 1406 rainbow trout collected from across the Colombia River basin (Micheletti et al., 2018). To assess the possible impact of hybridization with YCT, we also included whole‐genome sequencing data from 58 YCT individuals (R. Kovach and M. Campbell unpublished sequences).

### 2.3 DNA extraction, RAD library construction, and sequencing

DNA was extracted from fin clips using either Qiagen DNeasy tissue kits following the manufacturer's guidelines or a magnetic bead–based protocol (Ali et al., 2016). DNA was then quantified using Quant‐iT PicoGreen dsDNA Reagent (Thermo Fisher Scientific) with an FLx800 Fluorescence Reader (BioTek Instruments). SbfI Restriction site associated DNA (*RAD*) libraries were prepared with well and plate barcodes (Ali et al., 2016). The RAD libraries were then pooled, and capture was performed following the methods of Ali et al. (2016) and bait set (Ali et al., 2016; Kelson et al., 2019). The bait set targets approximately 500 SbfI RAD tags that are distributed across all chromosomes. The final RAD Capture (Rapture) library was sequenced with paired‐end 150‐bp reads on an Illumina HiSeq 4000.

Rapture sequencing data were demultiplexed by requiring a perfect barcode and partial restriction site match (Ali et al., 2016). Sequences were aligned to a recent rainbow trout genome assembly (Pearse et al., 2019) using the mem algorithm of Burrows‐Wheeler Aligner (Li & Durbin, 2009) with default parameters. SAMtools (Li et al., 2009) was used to sort, remove PCR duplicates, and count the number of alignments in binary alignment map files. A total of 1176 samples had approximately 10,000 or greater filtered alignments and were included in subsequent analyses. We followed the same protocol to align and filter the whole genome sequencing RBT and YCT samples from previous studies.

### 2.4 Hybrid detection

Because of the direct implications of hybridization and introgression for LCT conservation, we first performed a principal component analysis (PCA) as well an admixture analysis with all samples (individuals) to characterize potential rainbow and YCT ancestry. Due to biases associated with highly variable coverage among samples, we did not call genotypes for our PCA or admixture analyses. For the PCA, we instead used the Analysis of Next Generation Sequencing Data (ANGSD) software package (Korneliussen et al., 2014) to calculate a covariance matrix by sampling a single read per individual at each locus (Identity by State, or IBS sampling) with a minor allele frequency of at least 0.01 by adding the following parameters: minMaf 0.01 (minimum minor allele frequency), doCov 1 (compute a covariance matrix), doIBS 1 (sample one random read per locus), doMajorMinor 1 (define the major and minor alleles), minMapQ 20 (minimum mapping quality threshold), minQ 20 (minimum base quality threshold), SNP_pval 1e−12 (significance threshold for defining SNPs), GL 1 (calculate genotype likelihoods), doMaf 1 (estimate the major and minor allele frequencies), and doCounts 1 (count bases at each position, needed for IBS sampling). Since the RBT and YCT samples from previous studies are whole genome sequence data rather than RADseq data, and therefore are sequenced at many more genomic loci, we further restricted our PCA to those loci that passed our filters and were sequenced in more than 50% of individuals in our RADseq data alone. Final eigenvalue decomposition was performed using the prcomp function in R (RCoreTeam, 2020).

For admixture analysis, ANGSD was used to generate a beagle‐formatted genotype likelihood output file (Browning & Yu, 2009) by adding the doGlf 2 (create a genotype likelihood file using the GATK approach) parameter. Final admixture proportion estimation was performed with ngsAdmix (Skotte et al., 2013) by specifying three ancestral populations (*K* = 3) assumed to represent the three parental species or subspecies (rainbow trout and Yellowstone and LCT, see Sanz et al., 2009; Vaha & Primmer, 2006) As before, we restricted the loci we used to those passing our filters in the RADseq data alone. While we used *K* = 3 to allow individual ancestry to be partitioned into LCT, RBT, and YCT clusters, we also explored *K* = 4–9 to evaluate the possibility that low levels of assumed admixture could actually reflect natural structure within LCT populations (Shafer et al., 2015) rather than a small portion of non‐native trout ancestry within individuals. If the low level of non‐native ancestry identified in the LCT field samples was due to population structure within the LCT samples, we would not expect the proportion of non‐native ancestry to be the same at higher *K* values, since this variation would instead be assigned to the newly available clusters. This was not the case: across all *K* values assignments to the clusters containing the known RBT and YCT individuals and thus assumed to represent RBT or YCT ancestry were extremely similar (for example, the correlation for assignment probabilities to these clusters using *K* = 3 and *K* = 4 was >0.99 when using all samples, and 0.93 when considering only those with low levels of assignment (<1%) to these clusters, where we might most expect to see cluster switching). Accordingly, we used *K* = 3 for our interpretation of introgression in LCT samples. Since both the IBS PCA method implemented in ANGSD and ngsAdmix are designed to work with low sequencing coverage, these analyses were not restricted to bait‐targeted RAD tags, resulting in 43,263 SNPs contributing to both the PCA and admixture analyses. Although Rapture enriches for bait‐targeted loci, a significant proportion of reads still come from off‐target loci (Ali et al., 2016). These low‐coverage loci provide useful information for low‐coverage analysis methods (i.e., methods that do not require called genotypes). We removed all individuals with <99% LCT ancestry from further analyses.

### 2.5 Lahontan cutthroat trout SNP discovery and genotyping

After removing individuals with <99% LCT ancestry, SNP discovery and genotype calling was performed on the remaining samples using ANGSD with the same general parameters as above as well as doGeno 4 (call genotypes), doPost 2 (calculate genotype posterior probabilities), and postCutoff 0.95 (genotype posterior probability cutoff). Furthermore, since genotype calling requires higher sequencing coverage and off‐target loci are usually sequenced at a much lower depth, this analysis was restricted to sites from the bait‐targeted RAD tags (using the rf flag). These quality filtering criteria resulted in 897 SNPs out of 60,589 total interrogated genomic sites. Using the snpR package (Hemstrom & Jones, 2021), SNPs were filtered to remove loci not in Hardy‐Weinburg Equilibrium (HWE, *p‐*value of <1 × 10^−6^) in any set of samples collected from a given stream in a given year using an exact test (Wigginton et al., 2005). This filtering led to the removal of four loci, resulting in a final set of 893 SNPs. We also ran all analyses below after removing additional SNPs violating HWE across all samples (globally) as well as with all loci included (i.e., not removing any); neither approach led to any substantial change in our results (data not shown).

### 2.6 Estimation of *N*_e_ and genetic diversity metrics

Using our final marker set of 893 SNPs we first estimated effective population size (*N*_e_), one of the most important indicators of evolutionary potential (Waples, 2022) and population viability or, conversely, extinction risk (Antao et al., 2010; Luikart et al., 2010). However, because of several short comings of our final dataset we interpreted results of *N*_e_ cautiously as a general indicator of long‐term evolutionary potential but ultimately did not include *N*_e_ when evaluating concordance between genetic metrics and the MPVA abundance and extinction risk estimates. First, because our samples comprised mixed ages they violate the assumption of discrete generations in *N*_e_ models and are thus likely biased downwards due to a Wahlund effect arising from overlapping cohorts (Waples et al., 2014). As well, though in some cases they represent all fish found by agency managers, many of our sample sizes are far smaller than desirable for *N*_e_ estimation which may further push *N*_e_ estimates downward (Ackerman et al., 2017). We explore our interpretation of *N*_e_ estimates in light of these shortcomings and in the context of previous assessments of *N*_e_ in LCT with much larger sample sizes in the Discussion. We calculated *N*_e_ with two distinctive approaches. We first employed the NeEstimator software version 2.1 (Do et al., 2014) based on the linkage disequilibrium (LD) estimation approach (LDNE), which can be powerful in detecting population declines (Luikart et al., 2010) but can fail to provide estimates of population sizes when sample sizes are small or maintain little genetic diversity (Bernos & Fraser, 2016). We used jackknifing to generate confidence intervals, restricted the linkage analysis to loci on different chromosomes, and relied on pcrits (minimum minor allele frequencies) of 0.01 and 0.05. We also estimated the effective population size via sibship estimation and pedigree/family structure analysis using the COLONY software (Jones & Wang, 2010); here we assumed random mating using the FPLS likelihood estimation method, no sibship size prior, a medium run length and maximum likelihood precision, allele frequency updates during sibship estimation, no assumed monogamy, allowing for inbreeding, and a genotyping error rate of 0.01.

We then estimated several commonly‐used metrics of population genetic/genomic diversity (Ørsted et al., 2019; Schwartz et al., 2006) to assess genetic variability within LCT populations as indicators of conservation status and for comparison with MPVA results of abundance and extinction risk. The snpR R package was used to calculate average nucleotide diversity (*π*), observed heterozygosity (*h*_o_), Watterson's *θ* (*θ*_W_), Tajima's *θ* (*θ*_T_), the proportion of polymorphic SNPs (out of the 893 loci that were polymorphic in any population), and *θ* skew ([*θ*_T_ − *θ*_W_]/[(*θ*_T_ + *θ*_W_)/2]) (Peek et al., 2021) for each sampled stream/year combination. We estimated both Watterson's *θ* (Watterson, 1975) and Tajima's *θ* (Tajima, 1983) because these measures quantify different aspects of sequence diversity: Watterson's *θ* measures the number of segregating sites without considering allele frequencies, whereas Tajima's *θ* estimates polymorphism *and* frequency via the average number of pair‐wise differences between sequences. Accordingly, the two metrics should be equal under equilibrium processes, but strongly positive or negative values of the difference between the two (i.e., Tajima's *D*) can be indicative of nonequilibrium processes (Fischer et al., 2006; Fraik et al., 2021; Tajima, 1989). However, since Tajima's *D* is a measure of significance and is thus biased towards 0 in small sample sizes, we instead used the simple skew between the two variables (*θ* skew) to examine how divergence between them changes as overall genetic diversity changes.

### 2.7 Comparison of genetic metrics to MPVA‐generated abundance and extinction probability

As noted above, MPVA is a Bayesian hierarchical modeling framework that predicts abundance and extinction probabilities across all populations simultaneously, informed by spatio‐temporal environmental covariates and accounting for observation and sampling error; uniquely, it draws on information from well‐sampled populations to estimate extinction risk in less sampled populations (Leasure et al., 2019; Wenger et al., 2017). It includes: (1) an observation model that predicts site‐level abundance using all available field sampling data, (2) a sampling model that expands site‐level abundance to an estimate of population abundance (for years spanning when fish surveys occurred, see below), and (3) a population dynamics model used to project population abundances over time and estimate 30‐year extinction probabilities for each population (Leasure et al., 2019). We used outputs from the MPVA model applied to LCT (Neville et al., 2019) and fit a suite of linear models evaluating correlations with the different genetic diversity metrics (excluding *N*_e_ from these analyses as noted above). We expected populations with higher genetic diversity (based on estimates of *π*, *H*_o_, Watterson's and Tajima's *θ*, and theta skew) to have higher abundance estimates and lower 30‐year extinction probabilities. To test this hypothesis, we fit a series of a linear mixed‐effect models with each genetic statistic above as a fixed effect explaining MPVA extinction and the harmonic mean abundance estimates (see below), with a nested random effect of creek within basin where possible as a covariate. Where these models failed to converge, we modeled the genetic metric as a fixed effect with no random effects. These comparisons included a subset of our samples, i.e., only conservation populations (prioritized by management agencies for recovery due in part to an assumed lack of hybridization, USFWS, 2009) that are relict, or nonreintroduced. As noted above MPVA generated 30‐year extinction probabilities for all populations (with temporal genetic samples from the same stream thus having the same extinction estimates) but it estimated annual abundances only between the first and last year for which field sampling data were available for a given population (see Neville et al., 2019). Harmonic means thus were bounded by the first and either last year of MPVA sampling or the year of the genetic collection if that preceded the last demographic sample (see Table S1). Since the years for which we had MPVA data did not always match the years where we had genetic data, we repeated our analysis with only those stream/year combinations where the genetic collection was within 5 years of the years spanned in abundance estimation and therefore our MPVA harmonic mean abundances, without any substantial change to our results; we also evaluated these relationships using the abundance estimate from the year closest to the genetic sampling event (instead of the harmonic mean), with and without those greater than 5 years apart, with no change in results (data not shown). To reduce the chance of type II error, we used Holm's sequential Bonferroni (Holm, 1979) approach to adjust *p*‐values to correct for multiple comparisons.

Because these models showed little relationship between genetic and MPVA metrics (see Section 3), we explored random forest modeling (Breiman, 2001) as a flexible means of characterizing relationships between the multiple genetic metrics and the outputs of the population viability analysis. This machine learning approach incorporates various forms of information to construct and evaluate large sets of decision trees, undertaking a training process using random sampling of observed data and presenting the weighted mean prediction of the individual trees. We used this approach to determine if any combination of genetic variables or their interactions had any ability to predict demographic outcomes without explicitly creating a large number of models with predefined structure. Two random forest models were constructed using the ranger R package (Copeland et al., 2017), one for MPVA harmonic mean abundance and a second for 30‐year extinction probability estimates as response variables. In both cases, sampling location and all genetic metrics (excluding *N*_e_) were used as possible independent variables.

### 2.8 Evaluation of genetic erosion and association with MPVA estimates

Not only should standing levels of genetic variation be informative about population status but losses of diversity over time, or genetic erosion (Hoban et al., 2014), can provide further insight about risk (conforming as well to the true meaning of genetic monitoring as having a temporal dimension, Schwartz et al., 2006). We evaluated loss of genetic diversity over time by comparing values of genetic metrics (again excluding *N*_e_) in samples collected from the same population in different years. Additionally, to determine if temporal changes in genetic diversity were associated with among‐population differences in 30‐year extinction risk or changes over time in estimated harmonic mean abundance, we constructed simple linear regressions between these MPVA estimates and the per‐year average change in each of our diversity metrics for populations where we had both MPVA estimates and multiple years of genetic data (*n* = 12). We did not use any random effects for these models due to the small number of samples with both MVPA estimates and multiple years of genetic data. We again used Holm's (1979) approach to correct for multiple comparisons.

## 3 RESULTS

### 3.1 Localized hybridization observed via clustering analysis and PCA

**FIGURE 2:**
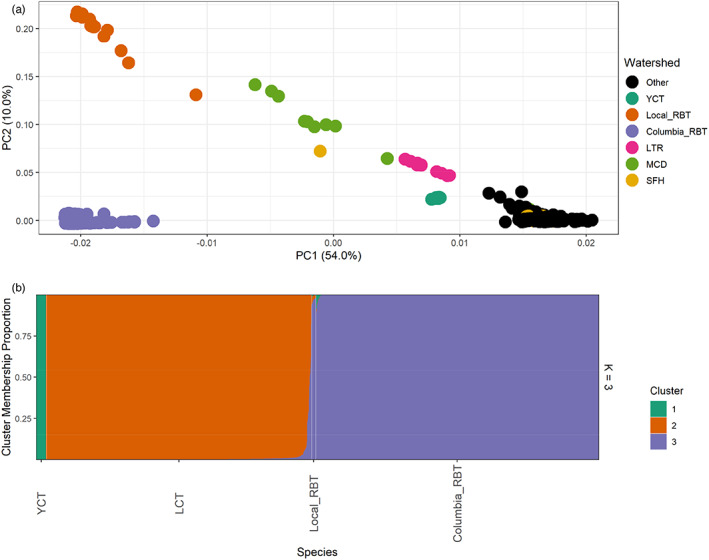
(a) Principal Components Analysis of full dataset of individual LCT collected in the field as well as naturalized rainbow trout from the Truckee River, California and Nevada (Local_RBT, orange dots); 1406 rainbow trout collected from across the Columbia River basin (Micheletti et al., 2018, Columbia_RBT, purple dots), individuals previously assessed as LCT‐RBT hybrids (MCD, lime green dots); and 58 YCT individuals (YCT, green dots), see text for further description. Lahontan watersheds containing individuals with notable hybridization detailed in the text are colored by major basin (South Fork Humboldt = SFH, gold, and Little Truckee River = LTR, pink); all others are shown in black (Other). (b) Results of admixture analysis using the clustering approach in ngsAdmix; each vertical bar represents an individual fish, with colors within indicating membership proportion in each of three clusters (*k* = 3). Along the *x*‐axis, YCT contains the YCT samples, represented in green; LCT contains the LCT samples largely represented in orange, with individuals at right demonstrating some hybridization with mostly RBT (purple); Local_RBT and Columbia_RBT contain the Truckee River naturalized rainbow trout and the 1406 rainbow trout collected from across the Columbia River basin (purple, Micheletti et al., 2018).

PC1 explained 54% of the variance in the PCA and clearly distinguished RBT, the most distantly related of the two non‐native trout, from most LCT samples, and was highly correlated with estimated RBT ancestry (*r* ^2^ = 0.998 for a correlation between the PC1 values and ancestry in the rainbow trout cluster from the admixture analysis). While most individuals were clustered along a continuous distribution of large values of PC1 (Figure 2), estimated to have high LCT ancestry and little to no RBT ancestry (Figure 2), a subset of individuals (*n* = 106) fell closer to the non‐native trout samples in the PCA and had more than 1% hybrid ancestry in the clustering analysis when combining both RBT and YCT ancestry. The majority of these individuals had mostly RBT hybrid ancestry (mean RBT and YCT ancestry = 9.1% and 1.5%, respectively). Beyond this, however, a handful of individuals had much higher hybridization levels, with a clear break at about 20% LCT‐RBT hybrid ancestry. Strikingly, in addition to the Truckee River and Columbia River reference RBT samples (orange ‘Local_RBT’ and purple ‘Columbia_RBT’ in Figure 2) and known hybrid samples from Indian Creek in the McDermitt Creek basin (MCD, lime green in Figure 2), these outliers included nine samples from Independence Lake in the Little Truckee River (LTR, pink in Figure 2), a priority conservation population in the Sierra Nevada mountains of California. These nine individuals contrasted with 10 additional Independence Lake samples that were part of the presumably nonintrogressed (black) cluster on the right. Lastly, the outliers included one individual from Second Boulder Creek in the South Fork Humboldt River (SFH, gold in Figure 2). Individual patterns of %LCT from the clustering analysis can be visualized clearly in Figure 2, where the YCT (green), LCT (orange) and RBT (purple) clusters were quite distinctive, except for the noted individuals to the right of the LCT cluster with mixed ancestry (largely with RBT). All 106 individuals with >1% hybrid ancestry were removed from subsequent analyses (note the McDermitt basin sample is tallied here but not included in Table 1 as it was not of interest beyond use as a verifier of known hybridization).

**TABLE 1:** Population sample characteristics, estimates of hybridization and genetic metrics, and demographic‐based estimates of abundance and extinction probability from a MPVA for LCT across their range in the interior western United States (see information below table and Table S1 for further details)

Basin	Creek	year	%LCT b	%LCT a	Finl n	*h*_o_	*π*	w.theta	t.theta	theta_d	ne.COL	ne.lcl	ne.ucl	Ext pr	HarMn
Black Rock	Colman	2016	100.0	100.0	18	6.0E−07	4.9E−07	1.8E−05	1.6E−05	−1.0E−01	26	13	57	–	–
Black Rock	Mahogany	2013	99.3	99.5	11	8.6E−07	7.6E−07	2.9E−05	2.6E−05	−1.1E−01	44	19	–	–	–
Carson	E Fk Crsn	2017	99.9	99.9	20	7.0E−07	5.6E−07	1.7E−05	1.9E−05	8.7E−02	17	9	38	–	–
Carson	Golden Cnyn	2017	99.9	99.9	12	7.5E−07	6.0E−07	1.8E−05	2.0E−05	1.1E−01	9	4	26	–	–
Carson	Murray Cnyn	2017	100.0	100.0	20	7.5E−07	5.7E−07	2.0E−05	1.9E−05	−5.7E−02	24	12	57	–	–
Carson	Poison Flat	2017	99.8	99.8	20	8.1E−07	7.0E−07	2.9E−05	2.4E−05	−1.8E−01	25	13	53	–	–
Coyote Lake	Whitehorse	2011	99.7	99.9	9	4.8E−07	3.8E−07	1.5E−05	1.3E−05	−1.8E−01	21	8	153	8.1	9143
Coyote Lake	Willow	1996	99.3	99.9	8	5.2E−07	4.3E−07	1.6E−05	1.4E−05	−8.5E−02	28	10	–	2.3	1797
E Fk Hmbldt	2nd Bldr	2016	95.1	100.0	15	8.0E−07	6.3E−07	2.2E−05	2.1E−05	−7.5E−02	16	8	36	21.8	52
E Fk Hmbldt	4th Bldr	2016	99.7	99.7	11	8.8E−07	6.9E−07	2.3E−05	2.2E−05	−4.0E−02	22	10	80	5.6	573
E Fk Hmbldt	N Fk Cold	2012	100.0	100.0	18	4.8E−07	3.9E−07	1.4E−05	1.3E−05	−8.1E−02	15	7	35	13.6	91
Lttle Hmblt	Abel	2016	99.8	99.8	19	6.5E−07	5.5E−07	2.8E−05	1.9E−05	−3.8E−01	36	21	88	7	168
Lttle Hmblt	Indian SF	2000	99.2	99.7	3	6.4E−07	6.1E−07	1.5E−05	1.4E−05	−7.3E−02	12	2	–	9.4	829
Lttle Hmblt	Indian SF	2013	99.5	99.5	2	6.1E−07	5.8E−07	9.9E−06	9.7E−06	−2.2E−02	–	1	–	9.4	177
Lttle Hmblt	Indian SF	2016	100.0	100.0	2	4.5E−07	4.2E−07	1.3E−05	1.5E−05	1.2E−01	–	1	–	9.4	177
Lttle Hmblt	Long Cnyn	2011	99.8	99.8	14	9.5E−07	1.1E−06	4.9E−05	4.1E−05	−1.7E−01	10	5	26	–	–
Lttle Hmblt	Long Cnyn	2016	98.9	99.6	2	7.5E−07	6.8E−07	1.9E−05	1.9E−05	−1.9E−02	–	1	–	–	–
Lttle Hmblt	SFLH Tribs	2010	99.9	99.9	30	7.2E−07	6.0E−07	2.3E−05	2.0E−05	−1.2E−01	29	16	54	7.5	3766
Maggie	Beaver	2009	98.6	100.0	11	6.4E−07	5.3E−07	1.8E−05	1.8E−05	2.0E−02	28	13	96	1.8	1655
Maggie	Beaver	2015	99.9	99.9	20	8.3E−07	7.0E−07	2.2E−05	2.3E−05	4.1E−02	26	14	53	1.8	1888
Maggie	Coyote	2009	99.7	99.8	13	7.9E−07	6.9E−07	2.2E−05	2.2E−05	−3.5E−02	13	6	35	3.4	1144
Maggie	Coyote	2015	99.8	99.8	18	7.4E−07	6.9E−07	2.4E−05	2.2E−05	−8.6E−02	44	23	112	3.4	1235
Maggie	Little Jack	2009	99.7	99.8	13	8.6E−07	7.8E−07	3.2E−05	2.6E−05	−1.8E−01	52	24	315	5.1	481
Maggie	Little Jack	2015	99.7	99.8	14	8.9E−07	7.4E−07	2.6E−05	2.7E−05	4.4E−02	11	6	30	5.1	526
Marys	Hanks	2012	99.9	99.9	22	8.6E−07	7.2E−07	2.4E−05	2.4E−05	−1.2E−02	17	9	34	76.9	49
Marys	Marys River	2016	99.9	99.9	39	7.5E−07	6.5E−07	3.0E−05	2.1E−05	−3.5E−01	22	14	40	0.3	7201
Marys	T	2002	99.9	99.9	9	6.8E−07	5.9E−07	1.9E−05	1.9E−05	−3.7E−03	36	15	–	0.1	3832
Marys	T	2017	99.8	99.9	17	6.5E−07	5.6E−07	2.0E−05	1.9E−05	−5.6E−02	30	16	66	0.1	3765
Marys	West Marys	2000	100.0	100.0	2	6.3E−07	6.5E−07	2.4E−05	2.3E−05	−6.3E−02	–	1	–	3	754
Marys	West Marys	2016	100.0	100.0	5	6.2E−07	5.6E−07	1.7E−05	1.8E−05	2.2E−02	20	7	–	3	657
Marys	Wildcat	2000	100.0	100.0	19	7.0E−07	6.0E−07	1.7E−05	1.9E−05	1.1E−01	22	11	52	42.7	1232
Marys	Wildcat	2017	99.9	99.9	20	7.1E−07	5.8E−07	2.1E−05	1.9E−05	−8.2E−02	12	6	28	42.7	268
N Fk Hmbldt	Foreman	1997	99.3	99.7	8	9.9E−07	9.1E−07	3.4E−05	2.9E−05	−1.4E−01	112	23	–	4	1462
N Fk Hmbldt	Foreman	2017	99.8	99.8	19	1.2E−06	1.0E−06	3.6E−05	3.4E−05	−5.3E−02	34	18	72	4	1093
N Fk Hmbldt	Gance	2000	99.7	99.8	6	1.0E−06	8.8E−07	2.0E−05	2.2E−05	9.0E−02	15	5	–	2.6	2255
N Fk Hmbldt	Gance	2017	99.9	100.0	18	7.6E−07	6.6E−07	2.1E−05	2.2E−05	6.6E−02	26	14	55	2.6	1282
Quinn	Andorno	2016	100.0	100.0	7	5.6E−07	5.1E−07	1.8E−05	1.9E−05	6.5E−02	21	9	–	–	–
Quinn	Corral Cnyn	2017	99.7	99.7	10	6.0E−07	5.0E−07	1.9E−05	1.7E−05	−1.3E−01	18	8	78	–	–
Quinn	Falls Cnyn	2017	92.6	NA	0	–	–	–	–	–	–	–	–	–	–
Quinn	Jackson	2017	94.5	NA	0	–	–	–	–	–	–	–	–	–	–
Quinn	Line Cnyn	2000	99.8	99.8	9	5.5E−07	4.5E−07	1.4E−05	1.5E−05	1.1E−01	24	9	270	21.1	1115
Quinn	Line Cnyn	2017	97.5	99.9	3	5.2E−07	4.6E−07	1.4E−05	1.5E−05	6.5E−02	–	1	–	–	–
Quinn	N Fk Battle	2013	100.0	100.0	8	6.7E−07	6.3E−07	2.4E−05	2.2E−05	−1.1E−01	56	18	–	–	–
Quinn	N Fk Battle	2016	100.0	100.0	7	5.5E−07	4.7E−07	1.5E−05	1.5E−05	4.0E−02	42	13	–	–	–
Quinn	Washburn	1997	99.7	99.7	18	5.8E−07	5.0E−07	2.4E−05	1.8E−05	−3.0E−01	31	16	68	14.8	288
Quinn	Washburn	2016	100.0	100.0	9	5.7E−07	4.7E−07	1.7E−05	1.7E−05	3.5E−03	24	9	19,601	14.8	322
Reese	Crane Cnyn	2009	100.0	100.0	21	2.8E−07	1.9E−07	9.5E−06	6.9E−06	−3.2E−01	5	2		–	–
Reese	Marysville	2016	99.9	99.9	5	4.2E−07	3.5E−07	1.2E−05	1.2E−05	−4.3E−04	–	1	–	–	–
Reese	Mohawk	2000	100.0	100.0	21	3.6E−07	2.8E−07	1.3E−05	9.7E−06	−2.6E−01	11	6	28	0.2	1250
Reese	Mohawk	2016	100.0	100.0	5	3.9E−07	2.8E−07	9.4E−06	9.7E−06	3.3E−02	20	5	–	–	–
Reese	Tierney	2001	100.0	100.0	16	6.8E−07	6.2E−07	2.1E−05	2.0E−05	−6.3E−02	34	18	110	72.5	54
Reese	Tierney	2012	100.0	100.0	3	5.2E−07	4.5E−07	1.5E−05	1.5E−05	1.4E−02	–	1	–	72.5	17
Rock	Frazier	1997	99.9	99.9	6	9.2E−07	7.7E−07	1.9E−05	2.1E−05	1.0E−01	15	6	–	24.2	3539
Rock	Frazier	2000	99.9	99.9	6	7.5E−07	6.5E−07	2.0E−05	2.1E−05	4.9E−02	60	14	–	24.2	3528
Rock	Frazier	2017	100.0	100.0	20	7.4E−07	6.1E−07	1.9E−05	2.0E−05	2.9E−02	17	9	38	24.2	986
Rock	Rock	2009	100.0	100.0	14	6.6E−07	5.9E−07	2.1E−05	2.1E−05	−1.6E−02	40	20	189	8.8	593
Rock	Rock	2017	100.0	100.0	8	6.3E−07	5.6E−07	1.9E−05	1.9E−05	2.1E−02	28	12	–	8.8	522
Rock	Toe Jam	2017	100.0	100.0	20	6.5E−07	5.5E−07	1.7E−05	1.8E−05	5.5E−02	33	18	76	5.1	1416
Rock	Will‐Nel	2009	99.9	99.9	20	7.4E−07	6.3E−07	2.1E−05	2.1E−05	1.7E−02	32	19	68	5.7	1151
Rock	Will‐Nel	2017	100.0	100.0	10	6.7E−07	5.6E−07	1.8E−05	1.8E−05	3.5E−02	36	15	351	5.7	1038
S Fk Hmbldt	Dixie	2011	99.8	99.9	6	8.2E−07	6.2E−07	1.8E−05	2.0E−05	1.2E−01	–	1	–	33.7	82
S Fk Hmbldt	Dixie	2014	99.9	99.9	3	8.0E−07	6.8E−07	1.4E−05	1.7E−05	1.6E−01	–	1	–	33.7	89
S Fk Hmbldt	Lee	2016	99.2	99.6	9	9.8E−07	9.0E−07	2.6E−05	3.0E−05	1.5E−01	24	10	230	–	–
S Fk Hmbldt	Long Cnyn	2012	100.0	100.0	5	5.5E−07	5.0E−07	1.8E−05	1.8E−05	−3.9E−02	–	1	–	2.1	931
S Fk Hmbldt	N Fk Green	2009	100.0	100.0	8	4.7E−07	3.6E−07	1.1E−05	1.2E−05	1.2E−01	19	7	–	–	–
S Fk Hmbldt	Pearl	2018	99.9	99.9	19	7.1E−07	6.2E−07	2.7E−05	2.1E−05	−2.3E−01	30	16	66	–	–
Steens OoB	Big Alvord	2000	100.0	100.0	3	2.7E−07	2.1E−07	6.9E−06	7.7E−06	1.0E−01	6	2	–	–	–
Steens OoB	Cottonwood	2000	99.9	99.9	15	3.0E−07	2.2E−07	1.1E−05	7.6E−06	−3.4E−01	10	4	35	–	–
Steens OoB	Little Alvord	2000	99.9	99.9	3	3.5E−07	2.7E−07	7.8E−06	9.3E−06	1.7E−01	6	2	–	–	–
Steens OoB	Mosquito	2000	99.7	99.9	4	3.3E−07	2.5E−07	8.7E−06	8.4E−06	−2.8E−02	12	3	–	–	–
Steens OoB	Pike	2000	100.0	100.0	2	3.0E−07	2.5E−07	8.1E−06	9.6E−06	1.7E−01	–	1	–	–	–
Truckee	Indpndnce L	2016	87.0	99.8	9	7.5E−07	6.7E−07	2.2E−05	2.2E−05	2.9E−02	144	37	–	–	–
Truckee	Meiss Mdws	2017	99.9	99.9	20	8.1E−07	7.3E−07	2.6E−05	2.5E−05	−5.2E−02	63	33	185	–	–
Truckee	Pole	2016	99.7	99.7	19	8.6E−07	7.4E−07	2.8E−05	2.5E−05	−1.3E−01	25	14	53	–	–
Walker	By‐Day	2015	99.8	99.9	53	8.7E−07	7.0E−07	3.8E−05	2.4E−05	−4.6E−01	16	9	34	–	–
Walker	Lower Wolf	2015	99.2	99.3	8	1.8E−06	1.5E−06	3.8E−05	3.9E−05	2.4E−02	16	6	80	–	–
Walker	Mill	2015	99.8	99.9	28	9.8E−07	7.2E−07	2.5E−05	2.2E−05	−1.3E−01	16	8	35	–	–
Walker	Mill	2017	100.0	100.0	18	5.6E−07	3.9E−07	1.2E−05	1.4E−05	1.0E−01	4	2	15	–	–
Walker	Murphy	2011	99.7	99.8	12	8.0E−07	6.0E−07	2.2E−05	2.0E−05	−6.6E−02	16	7	61	–	–
Walker	Silver	2015	100.0	100.0	7	8.8E−07	7.5E−07	2.2E−05	2.6E−05	1.6E−01	17	6	–	–	–
Walker	Slinkard	2015	99.9	99.9	27	9.6E−07	7.3E−07	2.2E−05	2.0E−05	−8.2E−02	26	15	48	–	–
Walker	Upper Wolf	2015	99.2	99.3	11	1.7E−06	1.4E−06	4.5E−05	4.8E−05	7.5E−02	24	11	79	–	–
Yuba OoB	Macklin	2016	100.0	100.0	20	7.7E−07	6.5E−07	2.1E−05	2.3E−05	6.4E−02	42	22	120	–	–
					Min	2.7E−07	1.9E−07	6.9E−06	6.9E−06	−4.6E−01	4	1	15	0	17
					Ave	7.1E−07	6.0E−07	2.1E−05	2.0E−05	−4.5E−02	27	10	456	14	1433
					Max	1.8E−06	1.5E−06	4.9E−05	4.8E−05	1.7E−01	144	37	19,601	77	9143

*Note*: For each population, we show the major basin, creek, year of genetic collection, % LCT before (b) and after (a) removal of hybrid individuals, final *n* (after removal of aforementioned individuals), homozygosity (*h*_o_), nucleotide diversity (*π*), Watterson's theta (w.theta), Tajima's theta (t.theta), theta skew (theta_d, see text), and effective population size estimates using COLONY (Ne.COL) with lower and upper confidence limits (ne.lcl, ne.ucl). Also given are estimates of 30‐year extinction probability (Ext pr) and the harmonic mean abundance (HarMn) generated from MPVA. Cells with “–” indicate where *N*_e_ estimates did not converge or data were not generated were not generated (i.e., MPVA estimates California, out of basin, transplanted or lake populations, e.g., see Neville et al., 2019). Minimum, Average and Maximum values shown at bottom.

### 3.2 Low *N*_e_ estimates, other metrics

Values for the various genetic metrics estimated with snpR are presented in Table 1, with minimum, maximum and average values for each metric at the bottom. LDNE successfully converged on bounded estimates of effective population size for only a small subset of the samples (1 for Ne0.05 and 4 for Ne0.01, Table S1); bounded estimates for the latter ranged from *N*_e_ = 2–30. In contrast, COLONY successfully produced converged point estimates for 53 samples ranging from 4 to 63 with an average *N*_e_ of 24 (Tables 1 and S1).

### 3.3 Genetic metrics show no correlation with either estimates of MPVA abundance or extinction probability

**FIGURE 3:**
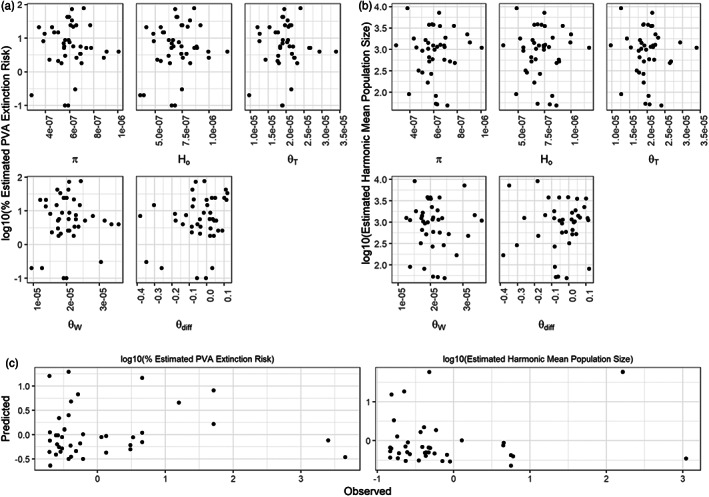
Comparison of estimates of various genetic diversity metrics with estimates of abundance and extinction probability generated from MPVA across nonhybridized LCT field populations meeting inclusion criteria (see text). Top 2 rows display results of linear models evaluating correlations between MPVA estimates of log10 30‐year Extinction (a, left three columns) and log10 Harmonic Mean Abundance (b, right three columns) versus nucleotide diversity (*π*), homozygosity (*H*_o_), Tajima's theta (Θ_T_), Watterson's theta (Θ_W_), and theta skew (see text, Θdiff). Bottom row (c) displays regression results from Random Forest Models of 30‐year Extinction (left) and Harmonic Mean Abundance (right) MPVA estimates (left and right panels, respectively) considering the above genetic metrics; observed value from data (*x*‐axis) versus weighted predicted tree values (*y*‐axis, see text for details).

Lahontan cutthroat trout populations were estimated to have a broad range of abundances and extinction probabilities (harmonic mean abundance: 17–9143 individuals, average 1433; probability of extinction: 0 to 77%, average 14%; Table 1). Forty‐one population samples (including some with temporal sampling) met inclusion criteria for our evaluation of the relationship between genetic diversity metrics and MPVA results (i.e., relict conservation populations of sufficient sample size with both genetic and MPVA data, Table 1). We observed no significant relationships between any of the individual genetic diversity metrics and either of the PVA statistics (extinction or harmonic mean abundance, Figure 3a,b; adjusted *p*‐values = 0.50–1.0 and 0.87–1, respectively, see Table S2). Likewise, jointly considering all genetic diversity metrics (except for *N*_e_) using Random Forest models yielded no predictive power and an extremely high error rate for out‐of‐bag samples for harmonic mean abundance or 30‐year extinction probability (Figure 3c, observed vs. predicted *r* ^2^ = <0; note that negative *r* ^2^ value here indicates no predictive power).

### 3.4 No evidence of genetic erosion observed over time

**FIGURE 4:**
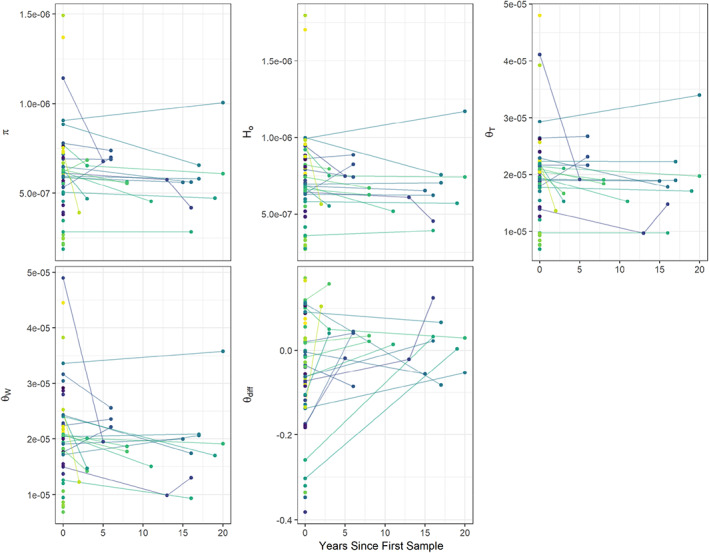
Difference in various genetic metrics over time for field nonhybridized populations of LCT that were temporally sampled (twenty populations, with the year span between sample periods ranging from 2 to 20 and averaging 12 years). Genetic metrics are nucleotide diversity (*π*), homozygosity (*h*_o_), Tajima's theta (Θ_T_), Watterson's theta (Θ_W_), and theta skew (Θdiff, see text).

**FIGURE 5:**
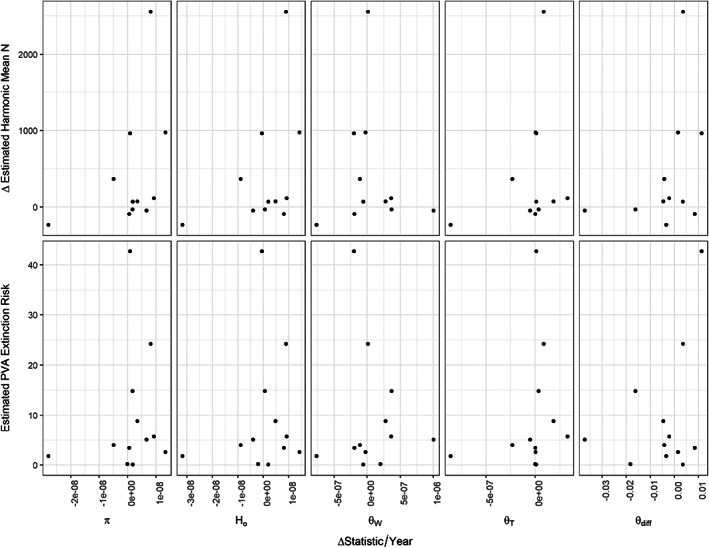
Evaluation of the relationship between change in estimates generated from a Multiple Population Viability Model of LCT versus the change in genetic estimates over time (Delta statistic/Year). For populations with temporal genetic sampling, change in harmonic mean abundance (Delta Estimated Harmonic Mean N, top panel) and 30‐year extinction probability (static probability, Estimated PVA Extinction Risk, lower panel) versus per‐year average change for each genetic diversity statistic: nucleotide diversity (*π*), homozygosity (*H*_o_), Watterson's theta (Θ_W_), Tajima's theta (Θ_T_), and theta skew (see text, Θ_diff_). See Table S1b for associated uncorrected and corrected *p* values.

Twenty populations had temporal sampling, with the span between sample periods ranging from 2 to 20 and averaging 12 years (Table 1). Genetic metrics did not change consistently across sampling locations from the first sampling period to the second (Figure 4). Furthermore, we observed no relationship between the average annual change in any of the genetic diversity metrics and MPVA estimated 30‐year extinction risk or changes in mean abundance estimates over time (Figure 5; Table S2b, corrected *p*‐values 0.78–1 and 0.28–0.75, respectively).

## 4 DISCUSSION

Our results highlight benefits of broad‐scale genetic assessment for a threatened species distributed across remote, difficult‐to‐sample watersheds, but also demonstrate several complexities of this approach. Drawing from population samples collected largely during standard agency sampling across this fish's range, we were able to characterize multiple aspects of the conservation status of LCT, most of which are concerning. For instance, introgression is a primary threat to the conservation of cutthroat and other trout species (Allendorf et al., 2001; Hohenlohe et al., 2013; Utter, 2004). Here, the inclusion of non‐native trout sequences and a simple initial clustering analysis allowed for evaluation of this threat across all LCT populations simultaneously and uncovered our first significant finding: that of substantially hybridized individuals in Independence Lake. As one of only two remaining native lake populations, which together occupy less than 1% of the historical lake habitat for LCT (USFWS, 2009), Independence Lake is a highly important conservation population and has been the focus of long‐standing conservation efforts. Our findings led to independent confirmation of these individual hybridization patterns using smaller sets of diagnostic markers (University of Nevada, Reno and California Department of Fish and Wildlife unpublished data) and intensive management of this issue to remove hybrids, a fortuitous option given the persistence of un‐hybridized individuals in the population (Allendorf et al., 2001; Neville & Dunham, 2011). They also prompted new assessments that have uncovered hybrids in several other populations and planning for more comprehensive field sampling to better monitor for hybridization throughout the LCT range in the future. These findings emphasize that researchers studying species where introgression is common or emerging may need to evaluate hybridization as a standing component of genetic monitoring (Peters et al., 2014). We strongly recommend this for studies of native trout given their long history of aquaculture and introduction world‐wide (Crawford & Muir, 2008; Kershner et al., 2019; Lever, 1996).

After removing hybrid individuals, our results suggest LCT populations have concerningly low levels of genetic variation overall. Evaluation of the quantitative values of genomic metrics themselves, for comparison to other studies for example, is difficult given different filtering and analytical methods (De la Cruz & Raska, 2014; Diaz‐Arce & Rodriguez‐Ezpeleta, 2019), but effective population size is a transferable metric of genetic status (Antao et al., 2010; Carroll et al., 2018; Leroy et al., 2018) that can provide an early warning about genetic risks (Hohenlohe et al., 2021; Olah et al., 2021) and with guidance on minimum sizes necessary for ensuring persistence and evolutionary capacity (Frankham et al., 2014; Franklin, 1980; Traill et al., 2010). However, because it can be difficult to measure accurately with whole population sampling such as ours (Serbezov et al., 2012; Waples et al., 2014), recent studies have demonstrated the benefit of the cohort‐based metric effective number of breeders (*N*_b_) as potentially useful for gaging abundance (Ferchaud et al., 2016; Luikart et al., 2020). In many respects, *N*_b_ would have been a more appropriate metric to evaluate for our eco‐contemporary questions even if high variability and unexplained variance in the relationship between *N*_b_ and census size (*N*_c_), particularly in small populations, has warranted caution when assuming a consistent relationship for monitoring changes in demographic attributes (Bernos & Fraser, 2016; Ferchaud et al., 2016; Ruzzante et al., 2016). Regardless, because estimating *N*_b_ requires relatively intensive, cohort‐targeted sampling it has been applied in few studies and on a handful of populations at a time (Kovach et al., 2020; Waples et al., 2018; Whiteley, Coombs, et al., 2015) and as is common to many real‐life situations (Waples et al., 2014) this degree of sampling was not feasible for our broad‐based assessment drawing largely on “found” agency‐collected and archived samples across a wide geography.

Recognizing the influences of small sample sizes and overlapping generations inherent to *N*_e_ estimation using our population samples (Ackerman et al., 2017; Wang, 2016; Waples et al., 2014), our *N*_e_ estimates were strikingly low—and yet notably they were highly similar to several previous assessments of LCT using microsatellites, much larger sample sizes, and a coalescent or linkage disequilibrium approach, respectively (*N*_e_ = 2–142 individuals, with sample sizes = 24–204 across Neville et al., 2006; Peacock & Dochtermann, 2012). In addition to this empirical grounding from earlier studies, it is possible to gage the degree of bias from the Wahlund effect from overlapping cohorts when using a linkage disequilibrium approach to *N*_e_ estimation, based simply on the ratio of adult lifespan/generation length (Waples et al., 2014); previous work on LCT suggests this ratio would be approximately 1.25 (See “*N*_e_ correction” in Supplementary information), meaning the influence of mixture LD would cause our estimates to fall somewhere from 70% to 90% of the actual *N*_e_ (see figure 6 in Waples et al., 2014). In some cases, our samples reflected the entire number of fish captured in the field after significant effort, so should not be biased by small sample sizes. If, however, we were additionally to account for the influence of small sample size generally by assuming further downward bias, even with a reasonably conservative approach of assuming our estimates are 25%–50% of the actual *N*_e_ our results would still be worrisome (Olah et al., 2021), especially given recent emphasis that effective sizes one to several orders of magnitude higher are needed to ensure long‐ and even short‐term persistence (see e.g., Frankham et al., 2014; Traill et al., 2010). Thus, even if substantially biased downward, our observed *N*_e_s raise concern about reduced evolutionary potential and an elevated risk of continued decline or extirpation for many LCT populations (Moyer et al., 2019; Newman & Pilson, 1997; Saccheri et al., 1998; Spielman et al., 2004). Improved sampling to refine *N*_e_ estimation for LCT may help resolve any need for more active consideration (Scott et al., 2020) or management of genetic diversity in the future (i.e., by assisted migration or re‐establishment of populations using multiple appropriate sources Fitzpatrick et al., 2016; Kovach et al., 2022; Robinson et al., 2017; Whiteley, Fitzpatrick, et al., 2015).

Despite these low effective population sizes and assumed associated low quantitative values of other metrics of diversity uncovered here, we still observed variation in these diversity estimates among all populations. Populations at the lower end of the diversity spectrum sustained only a fraction of the diversity in samples at the highest end (15% for *H*_o_, 13% for *π*, and 14% for both thetas). Though genetic metrics did not relate to abundance or extinction risk from our MPVA models (see below), a few notable patterns arose when considering all populations including transplanted or supplemented populations not evaluated in the MPVA comparisons. The very lowest values generally characterized transplanted populations that were established in the 1970s and 1980s outside of the historical range of LCT (in the Steens Mountains in Oregon) using small founder groups, as has been typical in the management of this fish (see Peacock et al., 2010), as well as several highly isolated conservation populations in the eastern and NW parts of the range (one thought to be have been extirpated more recently). Several populations on the higher end of the diversity spectrum included the remaining few with a known migratory life history (Neville et al., 2006, 2016). These findings emphasize the higher diversity harbored in interconnected populations (whether naturally or by assisted mixing, Kovach et al., 2022; Whiteley, Fitzpatrick, et al., 2015) and support continued management efforts to reestablish stronghold or meta‐populations to improve future resilience (Haak & Williams, 2012; Neville et al., 2016).

On the other hand, two populations considered demographic strongholds from the Coyote Lakes Basin in Oregon (Willow and Whitehorse Creeks) ranked at the lower end for several genetic metrics (one notably had an estimated *N*_e_ of 21 but the highest harmonic mean abundance estimate of all populations at 9143). Though relatively large for LCT in terms of stream miles, these streams have recently been found to be comprised of more marginal/intermittent habitat than previously appreciated (Gendaszek et al., 2020; Schultz et al., 2017) and likely represent highly dynamic habitat for the LCT within them. Another surprising result was that several populations from the Walker Basin ranked among the highest values for diversity metrics. All modern‐day Walker populations were founded with fish from By‐Day Creek, an LCT population discovered in the early 1900's that may be a relict but is of unconfirmed origin, and all were previously found to have very low genetic diversity compared with other LCT populations using microsatellite markers (heterozygosity values around 0.3, Peacock & Kirchoff, 2007). Persisting in the Sierra Nevada mountains of California, these populations reside in some of the best habitat currently available to LCT, which may help sustain diversity relative to other LCT populations in more desert‐like and volatile habitats. It is also possible that ascertainment bias in the microsatellites used previously, the development of which did not include Walker basin populations (Peacock et al., 2004), and/or genomic filtering (these data) are influencing these discrepancies to some degree, although different filtering protocols explored here produced highly correlated results (data not shown).

We additionally calculated *θ* skew (Peek et al., 2021), for which strongly negative values indicate an abundance of rare allelic variants and the potential influence of a non‐equilibrium process such as balancing selection or, more likely in this context, recent population expansion (Fraik et al., 2021). This metric can be sensitive to the timing of sampling relevant to bottlenecks or population expansion, and patterns for LCT were difficult to interpret in the end, although two populations targeted for intensive non‐native brook trout removal in the last decade were among those with the most negative values and may be benefiting from this management action.

Contrary to some other recent studies (Leigh et al., 2019; Leroy et al., 2018) there was no indication that genetic diversity declined consistently where we had temporal samples for direct measurement (see below). Additionally, neither our linear nor random forest models including genetic metrics demonstrated any ability to predict estimated abundances or 30‐year extinction probabilities from a recent MPVA (Neville et al., 2019). Based on the assumption that diversity metrics convey information about relative current population sizes (see empirical verification with allozymes in, e.g., Frankham, 1995), this result was somewhat surprising. We are confident in the overall results of the MPVA model, which incorporates all field sampling for LCT over several decades and includes relevant landscape‐scale covariates such as non‐native trout densities, stream temperature, and high flow magnitude; thus, it represents a uniquely comprehensive and data‐driven analysis framework (Leasure et al., 2019; Wenger et al., 2017) that draws on the most complete demographic data available for the species. However, even though results of MPVA showed broadly expected patterns, such as declining risk with increasing abundance and available stream habitat, estimated extinction risk varied greatly for small extents/populations in particular (see figure 2 in Neville et al., 2019) suggesting additional, unaccounted for factors may also be influencing demographic characteristics and risk in LCT. Further, though several studies in salmonids and other fishes have demonstrated promising relationships between genetic characteristics (*N*_e_ or *N*_b_, in particular) and abundance (Bernos & Fraser, 2016; Ferchaud et al., 2016; Ruzzante et al., 2016), the same and others (Ardren & Kapuscinski, 2003; Duong et al., 2013; Hargrove et al., 2022; Johnstone et al., 2013; Serbezov et al., 2012; Whiteley, Coombs, et al., 2015) have suggested high variation in reproductive success, life history differences, and localized and dynamic habitat features can drive variability in relationships between genetic metrics and abundances or extinction risk, even among neighboring populations (Belmar‐Lucero et al., 2012). For LCT, influences such as fire, persistent drought and stream intermittency (Gendaszek et al., 2020; Schultz et al., 2017), density‐dependent dynamics (Dunham & Vinyard, 1997), high population variability (Platts & Nelson, 1988), isolation (Dunham et al., 1997), and contemporary and historical metapopulation dynamics (Neville et al., 2006; Stearley & Smith, 2016) may have influenced genetic diversity (see, e.g., Blackman et al., 2021) to a degree that leaves little room for further genetic decline in some cases or correlation with demographic attributes overall. As our demographic MPVA model framework could not accommodate genetic characteristics (Neville et al., 2019) these findings may add an additional element of risk to be considered (Allendorf & Ryman, 2002; Frankham, 2005; Saccheri et al., 1998), as discussed below.

Other recent studies have been mixed in finding correlations between genetic/genomic and demographic metrics. Across a suite of desert lizards nucleotide diversity (*π*) was significantly associated with abundance and occupancy but explained only a small portion of variation (Grundler et al., 2019), while in amphibians genetic and field‐based measures of dispersal were highly correlated but genetic and field‐based estimates of abundance were not (once a strong outlier was removed, Wang & Shaffer, 2017). Evaluations based on IUCN Redlist status have shown that threatened species (classified as Critically Endangered, Endangered, and Vulnerable) as a whole maintained less genetic diversity than non‐threatened populations or those of Least Concern (Brüniche‐Olsen et al., 2019; Li et al., 2016) even though genetic metrics did not relate to the abundance of mature individuals (Willoughby et al., 2015); still others have found no association between genetic metrics and demographically‐based classification of extinction risk (Brüniche‐Olsen et al., 2018, which the authors note may point to a weakness in demographically‐derived conservation classifications, see below). Thus, despite long‐standing demonstration of correlations between genetic diversity and abundance (e.g., Frankham, 1995, and other citations above), the substantial variability observed here and in numerous other studies suggests verification and calibration of this relationship in context (e.g., Ferchaud et al., 2016) is warranted before characteristics of modern abundance can be assumed from genetic diversity metrics, or vice versa.

Undoubtedly, however, the established strength of genetic metrics is that they *do* capture influences other than time‐bound abundance estimates (Doyle et al., 2015) and reflect aggregate effects over long time frames (Araki et al., 2007; Duong et al., 2013). Thus, as noted above the low effective population sizes and relative genetic diversity observed here for many LCT populations reflects the cumulative history of these influences (Almeida‐Rocha et al., 2020; Reed & Frankham, 2003), particularly in the dynamic Great Basin environment (Platts & Nelson, 1988; Smith et al., 2002)—even where populations are observed to be quite abundant in modern sampling. We emphasize, therefore, that genetic metrics can indicate additional fitness and viability risk (Allentoft & O'Brien, 2010; DeWoody et al., 2021; Ørsted et al., 2019) beyond that predicted by purely demographic metrics; i.e., though the goal to use genetic metrics as an indicator of abundance did not play out in this study the incongruency observed here points to the distinctive yet complementary nature of these types of data (Belmar‐Lucero et al., 2012; Dunham et al., 1999; Hargrove et al., 2022). Where demographic information may be of immediate importance in understanding recent population dynamics and associated modern environmental influences, genetic metrics provide critical insight into fitness, future adaptive potential and extinction risk (DeWoody et al., 2021; Lande, 1988; Saccheri et al., 1998; Shafer et al., 2015) that is unattainable with demographic data alone. This complementarity has led many to stress the need to consider genetic/genomic information as essential to characterizing biodiversity (Proença et al., 2017; Reed & Frankham, 2003) and risk (Laikre, 2010) and an important component of status listing in addition to demographic metrics (Brüniche‐Olsen et al., 2018; Frankham et al., 2014; Willoughby et al., 2015), a recommendation with which we strongly agree.

## CONFLICT OF INTEREST

The authors declare no conflict of interest.

## Supporting information


Tables S1‐S2

Appendix S1

## Data Availability

All data are housed in github: https://github.com/hemstrow/N_H_etal_LCT.
