# Supplementary material for: Population genomic monitoring provides insight into conservation status but no correlation with demographic estimates of extinction risk in a threatened trout
Source: Evol Appl. 2022 Sep 4;15(9):1449–68. doi: 10.1111/eva.13473 (PMC9488680; doi:10.1111/eva.13473)
Supplement: Supplementary file 2 — Appendix S1 [file EVA-15-1449-s001.docx]

**Supplementary Tables**

**Table S1.** Similar to Table 1 (see descriptor), with population sample information and estimates of genetic and demographic characteristics from a Multiple Population Viability Analysis for Lahontan cutthroat trout populations across their range in the interior western United States, but presenting all data. For each population we show: major basin, creek, year of genetic collection, proportion of polymorphic loci, initial %LCT ancestry, %LCT after removal of individuals with <0.99%LCT, %ancestry for rainbow and Yellowstone cutthroat trout before and after removals, initial and final sample sizes (n), homozygosity (ho), nucleotide diversity (π), Tajima’s theta (ts.theta), Watterson’s theta (ws.theta), and theta skew (theta_diff, see text). Effective population size estimates given using LDNE for critical values of 0.05 and 0.01 (Ne_0.05 and _0.01) with lower and upper confidence limits for each (LDNe_lCIj and _uCIj ), and using COLONY (Ne_COL) with lower and upper confidence limits (ne.lcl and .ucl). Also given are estimates of extinction probability (PVA.Extinction), abundance (PVA.Abundance) and associated year, harmonic mean abundance (N_harmean) and the years over which it was calculated (year_start, year_end) generated from MPVA. Cells with “-“ indicate where Ne estimates did not converge or where MPVA estimates were not generated (California, out of basin, transplanted or lake populations e.g., see Neville et al. 2019).

**Table S2A.** Significance levels and sample size for linear models used to evaluate correlations between the different genetic statistics (except for Ne metrics, see text), and outputs of extinction probability and harmonic mean abundance from the MPVA models; p values given as both uncorrected (B, C) and adjusted (F, G) for multiple comparisons. See also Figure 3. **Table** **S2B** Significance levels for linear models used to evaluate correlations between extinction probability and change in harmonic mean abundance, and the different genetic statistics, as above. See also Figure 5.
